# Improving Health Care for Patients with Multimorbidity: A Mixed-Methods Study to Explore the Feasibility and Process of Aligning Scheduled Outpatient Appointments through Collaboration between Medical Specialties

**DOI:** 10.5334/ijic.6013

**Published:** 2022-03-01

**Authors:** Cathrine Bell, Charlotte Weiling Appel, Anne Frølich, Anders Prior, Peter Vedsted

**Affiliations:** 1 Diagnostic Centre – University Research Clinic for Innovative Patient Pathways, Silkeborg Regional Hospital, Department of Clinical Medicine, Aarhus University, DK; 2 Innovation and Research Centre for Multimorbidity, Slagelse Hospital, Region Zealand, Centre for General Practice, Faculty of Health and Medical Sciences, University of Copenhagen, DK; 3 Research Unit for General Practice, Aarhus, DK

**Keywords:** integrated care, ambulatory care, multimorbidity, outpatients, appointments and schedules, delivery of health care

## Abstract

**Background:**

Many patients with multimorbidity have appointments and parallel trajectories in several outpatient clinics across medical specialties. This organisation may disintegrate care and challenges the navigation of the healthcare system.

**Methods:**

This study explored the feasibility of an intervention targeting patients seen in several outpatient clinics for multiple diseases. The intervention aimed to coordinate outpatient appointments through enhanced collaboration across medical specialties. Feasibility and process were assessed through mixed methods by tracking the intervention through prospectively collected data and through semi-structured interviews with patients and healthcare professionals.

**Results:**

A multidisciplinary outpatient pathway was established as an intervention. Appointments for different medical specialties were scheduled on the same day, information was rapidly transferred to the receiving outpatient clinic, and a multidisciplinary conference resulted in the circulation of a joint summary. In the first year, 20% of eligible patients were enrolled. Appointments were aligned in 15% of patients, and blood samples were reduced by 29%. Overall, intervention components were delivered as intended and seemed acceptable, although the patient selection needed refinement.

**Conclusion:**

It seems feasible to set up an intervention for patients attending several hospital outpatient clinics. Future interventions should focus on selecting patients in greatest need for alignment of appointments.

## Background

Multimorbidity is considered a global healthcare challenge [1,2]. Healthcare systems are challenged by the increasing prevalence of patients with multimorbidity with complex healthcare needs. Multimorbidity is commonly defined as the co-existence of two or more chronic medical conditions in the same individual [3,4,5], and it presents several challenges.

Patients with complex multimorbidity commonly experience a high treatment burden in terms of understanding and adhering to care plans [6]. Engaging with different healthcare professionals and attending multiple appointments [7,8,9] may affect the patient’s quality of everyday life [6,10,11]. The different diseases tend to cluster and interact and managing health can become a difficult task for both the patients and for the healthcare professionals supporting them [10,12,13,14,15,16,17]. Higher numbers of concomitant conditions and polypharmacy, which adversely affect the patient’s well-being, increases the complexity of managing diseases [13,18]. The comprehensive needs for long-term care and support of people with multimorbidity place high pressure on the healthcare systems, as the utilisation of healthcare services increases by number of diseases [19,20].

Alleviating some of these factors through integrated care may optimise the trajectories for both patients and healthcare professionals [21], who are likely to benefit from well-coordinated integrated care [11,22,23,24,25,26]. Modern organisations are based on highly specialised healthcare that tends to focus on single disease. This ensures high professionalism in single-disease care, but also results in fragmented care, which is difficult to navigate [27]. As the care needs are complex, delivery processes involve numerous interfaces to support tailored approaches. Multimorbidity may require many different medical specialties to work together in a coordinated way [23,27,28].

Meeting the organisational challenges will require a stronger integration of the existing services, but the evidence on the approaches to integrated care is sparse. Most studies are conducted in primary care and community settings [20,29,30,31,32,33,34,35]. However, there is a paucity of research on hospital interventions and attempts to integrate or reduce the utilisation of hospital services [29,31,32,36,37], although innovative programs involving integrated care have been identified [38].

We developed a Multidisciplinary Outpatient Pathway (MOP) for multimorbid patients seen in outpatient clinics, aiming to integrate services provided by different medical specialties. This study aims to assess the feasibility of the intervention during the first year with a process evaluation.

## Methods

### Setting

The study was conducted at Silkeborg Regional Hospital in Denmark, a development hospital specialising in adaptation and creation of integrated patient pathways. It has a catchment area of 93,000 inhabitants. Through tax financing, the Danish healthcare system provides free universal access to public healthcare, including hospitals, general practice, and selected partners in primary and secondary care [39,40].

### Study design

This was a feasibility study with a process evaluation of a complex intervention tailored for multimorbid patients seen in the different outpatient medical clinics. The data was collected from 15 August 2018 to 14 August 2019 using convergent mixed methods. The study was guided by the frameworks of the Medical Research Council (MRC) on the development and evaluation and on process evaluations [41,42]. For reporting, the TIDieR checklist was followed [43,44].

### Development

Available evidence was reviewed on targeted care management for patients with multimorbidity [22,29,32,38,45,46,47]. To facilitate broad support and involvement in designing a care model, a co-creating participatory approach was applied [48,49,50]. Available details in Appendix 1 and 2 [45,51].

### Intervention theory

**Figure 1:**
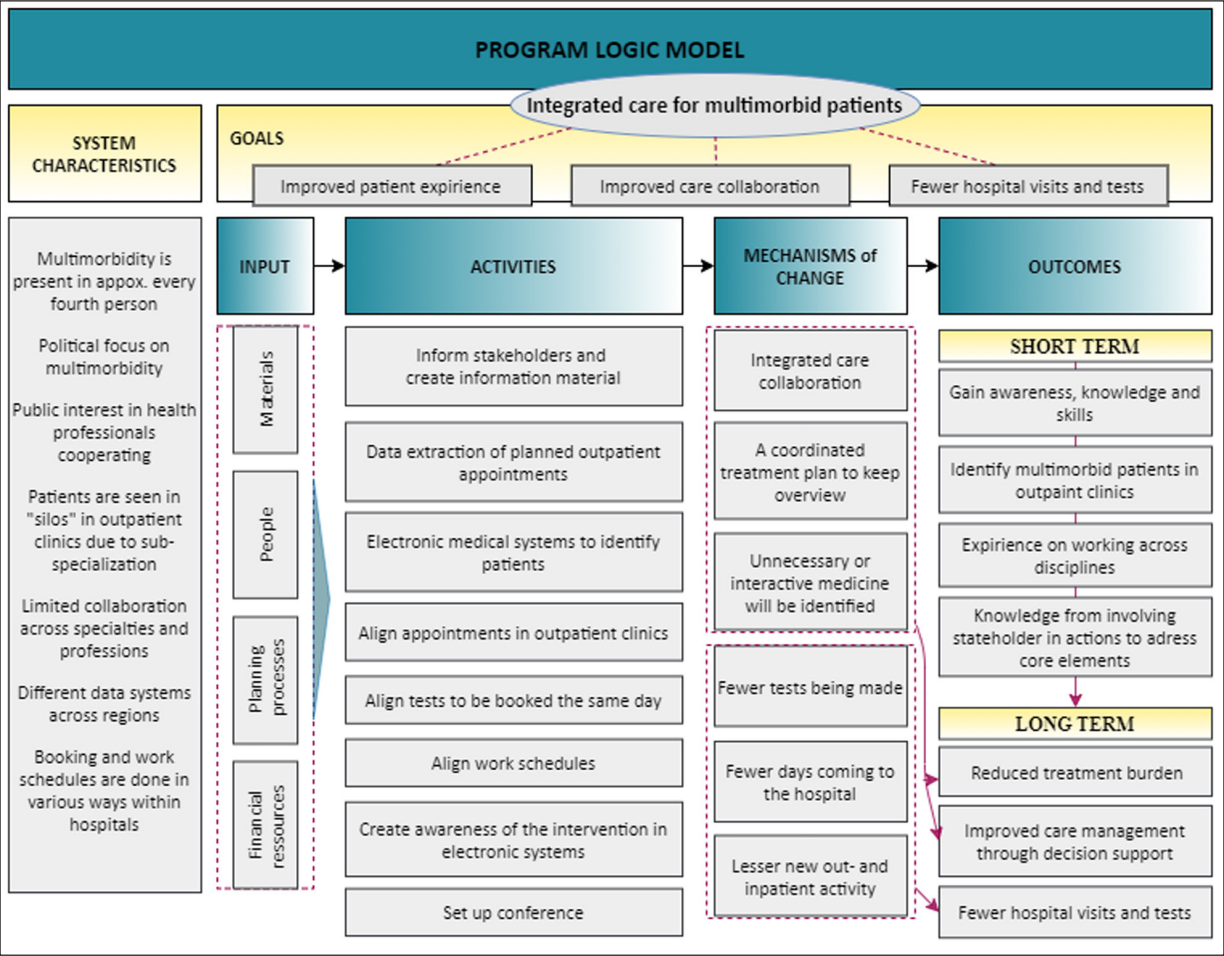
The program logic model.

The MRC framework recommends a clear description of the intervention, and how it is expected to work [42]. Logic modelling was used to synthesise and describe the complexity to help clarify causal assumptions [45] (***Figure 1***). For theoretical basis for reorganising health services, we were inspired by existing evidence, consulted stakeholders (Appendix 1), and drew elements of Wagner’s Chronic Care Model and the SELFIE framework.

### The intervention

**Figure 2:**
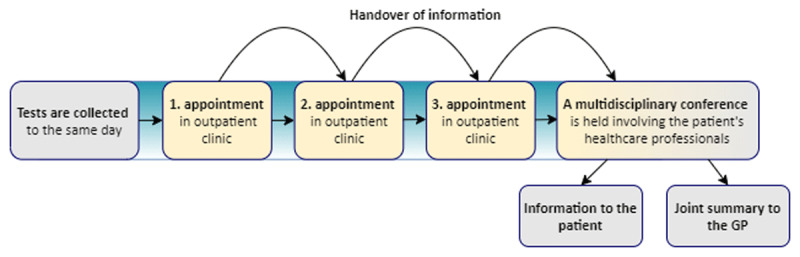
The Multidisciplinary Outpatient Pathway.

The MOP consisted of three elements (***Figure 2***). *First*, nurse care managers (experienced with coordinating care) received a monthly list of forthcoming patients and their medical appointments with nurses or physicians in outpatient clinics. Participants were identified proactively, as registered in the electronic health records [52]. Outpatient appointments were consecutively scheduled to take place on a certain date. The booking was arranged with the individual outpatient clinics. Schedules for care professionals were coordinated and time slots were reserved in appointment calendars and released fourteen days before the date of reservation, if not used. Laboratory personnel were made aware of the need to coordinate testing. *Second*, the delivering specialty wrote a summary with care-related information (rapidly passed) to the receiving clinic. *Third*, afterwards, the involved physicians and nurses attended a conference, resulting in a joint care plan with oral feedback and notice of modifications to the patient and a written summary to the GP. This was formed as a supplementary summary listing any modifications of the individual specialty summaries.

As new candidate appointments for alignment entered the monthly lists, previously ineligible appointments could re-enter. The intervention was launched as optional to patients. The program was refined progressively with weekly evaluations and adjustments during the first months.

### Participants and place

Data were extracted on outpatient appointments scheduled less than six weeks apart; this was done continuously every month. These data lists constituted the basis for potential candidates. We excluded appointments related to other research projects, new referrals for diagnosing purposes, and procedures conducted without a subsequent care appointment. Adult patients with two or more chronic conditions (duration for at least six months) with scheduled appointments in two or more medical outpatient clinics were included. The intervention was restricted to five specialties: cardiology, pulmonology, rheumatology, nephrology, and endocrinology. The selection was based on highest outpatient activity across specialities while considering the relevance for integration [1,53]. Existing outpatient clinics were used, and the intervention took place each week on the same two weekdays.

### Contextual factors

were considered, acting as barriers or facilitators to the implementation. The outpatient clinics had different ways of arranging appointments in the hospital’s booking system, and arrangements were made by different secretaries and nurses. Appointments were booked months in advance, leaving little opportunity to negotiate rescheduling. The alignment was challenged by; subspecialisation, patients were often followed by the same healthcare professionals, and a national strategy [54] mandated the allocation of a primarily responsible physician. The intervention needed to accommodate these factors. Outpatient clinics were located in proximity to each other, which facilitated to movement between clinics. At this hospital, staff were experienced in reorganising patient pathways.

### Patient characteristics and PROMS at baseline

Data on age, gender, civil status, distance to hospital from home address, diagnoses, and outpatient trajectories were retrieved through the Patient Administrative System (PAS). The Danish Multimorbidity Index [55], based on the International Classification of Diseases, 10^th^ revision, was used to estimate the frequency of diagnoses. Patients were sent a questionnaire before entering the MOP. This included the Short-Form Health Survey 12-item (SF-12) for assessment of health-related quality of life. It uses two weighted scales (mental and physical functioning) ranging from 0–100 (low-high)[56]. Care services were assessed through the 20-item Patient Assessment of Chronic Illness Care questionnaire (PACIC), which considers whether patients receive patient-centred care. Response categories range from 1–5 (never to always) aggregated into an overall score and five subscales: patient activation, delivery system design/decision support, goal setting/tailoring, problem solving/contextual and coordination/follow-up. Higher scores indicate high quality of care [57]. Also, the 10-item Multimorbidity Treatment Burden Questionnaire (MTBQ), which consists of five response categories; presented as a global score grouped into four categories, scores ranging from 0 (no burden) to ≥ 22 (high burden)[58] (***Table 1***).

**Table 1:** Characteristics of patients in the Multidisciplinary Outpatient Pathway from 15 August 2018 to 14 August 2019.


			N	%

**Patients**			102	100

**Age, years**	median = 71 years, IQR (63; 76)			

<60			19	18.6

60–69			27	26.5

70–79			42	41.2

80–89			14	13.7

**Gender**, female			46	45.1

**Distance to hospital, km**	median = 7.7 km, IQR (3.7; 17.8)			

<10			52	56.5

10–19			23	25.0

20<			17	18.5

**Civil status**				

Living with a partner			54	52.9

Not living partner			39	38.2

**Chronic diseases**	median per patient = 5 diagnoses, IQR (3; 7)		491	100

Hypertension			50	10.2

Diabetes mellitus			47	9.6

Chronic obstructive pulmonary disease			45	9.2

Dyslipidaemia			38	7.7

Allergy			36	7.3

Ischemic heart disease			35	7.1

**Concomitant chronic diseases** ^1^				

Hypertension – Dyslipidaemia			34	33.7

Hypertension – Diabetes mellitus			30	29.7

Chronic obstructive pulmonary disease – Hypertension			26	25.7

**Well-being**				

Physical health, summary score SF12		mean score = 31.7	47	46.1

Mental health, summary score SF12		mean score = 46.1	47	46.1

**Trajectories in outpatient clinics**			239	100

Pulmonology			68	28.5

Cardiology			45	18.8

Endocrinology			58	24.3

Rheumatology			36	15.1

Nephrology			20	8.4

Other non-included outpatient clinics			12	5.0

**Trajectories per patient** ^2^			239	100

2			75	73.5

3			21	20.6

4+			6	5.9

**Patient assessment of chronic integrated care**	**mean**	**95% CI**		

Patient activation	3.3	(2.94; 3.57)	65	63.7

Delivery system design/decision support	3.6	(3.33; 3.78)	64	62.8

Goal setting	2.8	(2.58; 3.04)	65	63.7

Problem-solving/Contextual counselling	3.1	(2.81; 3.36)	62	60.8

Follow-up/Coordination	2.4	(2.14; 2.69)	63	61.8

Overall score	2.9	(2.23; 3.64)	61	59.8

**Multimorbidity treatment burden**	median overall score = 7.5, IQR (2.5; 15)			

No burden (score 0)			9	19.6

Low burden (score <10)			17	37.0

Medium burden (score 10–22)			17	37.0

High burden (score ≥ 22)			3	6.5


^1^ Most frequent among diagnoses appearing in the Danish Multimorbidity Index. ^2^ Including non-included outpatient clinics other than the five clinics. Numbers may not add to 100% due to missing values.

### Quantitative implementation assessment

Reach was assessed through participation. Scheduled appointments were monitored by the care managers, who facilitated sequential appointments in agreement with the patient and clinics. From PAS, we retrieved dates for blood sampling in the fourteen days leading up to the appointment date to assess if samples had been collected.

Fidelity: The care managers documented when and why aligning appointments was practicable or not, if patients declined, cancellations, and if the conference was conducted. The time interval between appointments, (from end of dictation summary to start of next appointment), included PAS data on transcript times from physicians in all specialties and nurses in the endocrinology outpatient clinics. PAS data also included when a joint summery was transferred to the GP.

### Qualitative assessment: Experiences with the MOP

*Acceptability and fidelity*: Individual interviews were conducted with ten patients and ten healthcare professionals on their experiences of the intervention, including uncertain aspects of program design and delivery [45,49]. Respondents were recruited at 2–4 days after entering the intervention in chronological order of presentation. A semi-structured interview guide was used; one for patients and another for healthcare professionals [59]. Details were elicited by open questions and at the end of each interview, the interviewer summarised key features and allowed the respondent to elaborate and validate. Interviews were audio-recorded and transcribed verbatim. Using Malterud’s systematic text condensation strategy, CB and CWA analysed the transcripts separately for healthcare professionals and patients [60]. This includes four steps of analytic process: 1) total impression – from chaos to themes, 2) identifying and sorting meaning units – from themes to codes, 3) condensation – from code to meaning, and 4) synthesising – from condensation to descriptions and concepts.

## Results

### Study population

In the first intervention year, 102 patients received the intervention (***Table 1***). They were aged 71 years (median), 46% were females, 54% lived with a partner, and the median distance to the hospital was 7.7 kilometres (4.8 miles). The patients had a median of five diagnoses, with hypertension and diabetes mellitus being the most frequent. The patients scored an average of 32 points on the physical scale and of 42 points on the mental health of the SF-12. The quality of care was assessed to an average of 2.9 on the PACIC questionnaire. About 43% of the patients reported treatment burden to be medium/high. The vast majority (94%) had 2–3 trajectories in outpatient clinics.

### Reach

From the care managers’ lists for potential alignment, 2027 unique appointments (100%) were assessed. As the dataset comprised many lists of appointments retrieved every month, some appointments reappeared in newer lists as new appointments created new combinations for alignment. Therefore, 2367 appointments (116.8%) were evaluated in total. The intervention reached 102 patients, who comprised a 20% inclusion, and 309 appointments (15.2%) were coordinated. In median, patients entered the MOP once (interquartile range (IQR); 1: 4) and had two appointments coordinated (IQR; 2:4). Reasons when alignment was not possible are shown in ***Table 2***. Blood sampling was requested 142 times by different specialties in 80 patients. The number of times that a patient was sampled for blood was reduced by 29.1% to 101 blood samplings. Two requisitions from different specialties were taken simultaneously in 33 cases, and three requisitions were taken simultaneously in four cases.

**Table 2:** The Multidisciplinary Outpatient Pathway (15 August 2018 – 14 August 2019).


ELIGIBILITY – ALL ASSESSED APPOINTMENTS APPEARING ON THE COORDINATORS’ LISTS	N	%

Patients	510	100.0

Appointments assessed by care managers incl. repeaters^1^	2367	116.8

Unique appointments^2^	2027	100.0

Unique appointments/patient	median = 3	IQR (2; 5)

Unique appointments by same specialty^3^	1900	93.7

**INTERVENTION APPLIED**		

Multidisciplinary outpatient pathway [Intension to treat]	131	

Patients with intervention	102	20

Multidisciplinary outpatient pathway per patient	median = 1	min: max (1; 4)

Coordinated appointments	309	15.2

Coordinated appointments per patient	median = 2	IQR (2; 4)

Coordinated appointments per specialty	268	13.2

Specialty involvement	268	100

Pulmonology	86	32.1

Rheumatology	43	16.0

Cardiology	39	14.6

Endocrinology	76	28.4

Nephrology	24	9.0

Coordinated appointments by specialty per patient	median = 2	IQR (2; 3)

Healthcare professionals coordinated for conference	267	100

Nurses	70	26.2

Physicians	197	73.8

Reduction in blood sample collection	41	29.1

**REASONS WHY COORDINATION WAS NOT POSSIBLE** ^4^		

Appointments not coordinated for intervention	1718	84.8

Due to the other appointment(s)	295	14.6

Relevant healthcare professional not present	280	13.8

No available time slots/timely coordination not made	199	9.8

Appointments already coordinated	157	7.8

Work schedule not ready	135	6.7

Treatment/clinical cause	131	6.5

Other reasons, e.g. cancellation	38	1.9

**TRACKING FIDELITY**		

Multidisciplinary outpatient pathway [Intension to treat]	131	100

Full intervention with conference held	109	83.2

Notification sent to general practice after conference	104	95.4

Declined by patient	4	3.1

Cancelation by patient/no show	6	4.6

Pathway without conference being held	12	9.2

Dictation summary for receiving specialty	88	

Ready	29	33.0

Not ready	59	67.1

Patient wait time between specialties, minutes	median = 35	IQR (19; 54)


^1^ Appointments may reappear on the care managers’ lists due to new combination possibilities in cross-sections. ^2^ All possible appointments made by the coordinators, including times when the intervention was only partially received. ^3^ Within same specialty, patients may have had a nurse’s appointment followed by a doctor’s appointment on the same day. ^4^ Out of the 2367 appointments, documented by the care managers.

### Fidelity

Often, lack of flexibility in other appointments was the reason why coordination was not possible. When it was possible to coordinate appointments, few patients declined entering the pathway (3.1%). There were few cancellations (4.9%). The intervention was predominantly conducted according to the protocol (***Table 2***), which was supported by interviews. However, the dictation summaries were largely unavailable for the receiving outpatient clinic before consultation start. The median waiting time between appointments was 35 minutes (IQR 19:54) for the patients. The full intervention was held 83.2% of the time, with a joint summary transferred to general practice in 95.4% of cases.

### Experiences through interviews

Respondents’ characteristics are reported in ***Table 3***. The interview duration was in median 26 minutes (IQR 21:35) and 30 minutes (IQR 26:40) for healthcare professionals and patients, respectively.

**Table 3:** Characteristics of healthcare professionals and patients interviewed on their experience with the Multidisciplinary Outpatient Pathway.


HEALTHCARE PROFESSIONALS^1^	N = 10	

**Median age**, years	56	(IQR, 47:58)

**Gender**, female	5	

**Specialties**		

Cardiology	2	

Endocrinology	3	

Pulmonology	2	

Nephology	1	

Rheumatology	2	

**Nurses**	3	

Years since nursing authorisation (median)	34	(IQR, 17:34)

**Physicians**	7	

Years since specialty authorisation (median)	12	(IQR, 7:14)

**PATIENTS** ^2^	N = 10	

**Median age**, years	75	(IQR, 71:82)

**Gender**, female	2	

**Educational level** ^3^		

>10 years	3	

10–15 years	5	

<15 years	2	

**Civil status**		

Living with partner	6	

Living alone	4	

**Speciality trajectories in outpatient clinics**		

Cardiology	5	

Endocrinology	4	

Pulmonology	6	

Nephology	4	

Rheumatology	4	


^1^ Data available at *autregweb.sst.dk*. ^2^ Data collected through the interviews. ^3^ According to UNESCO’s International Standard Classification of Education (ISCED) 2011. IQR = interquartile range.

#### Healthcare professionals

##### Theme 1) Handover of information

A few stated that they did not read the transferred summary from the delivering specialty. Also, it was not always ready in due time for the receiving specialty. Some experienced that the summary should be more focused towards a multidisciplinary collaboration and easier to interpret, which could require more efforts to be invested in making a summary. This meant going beyond the usual way of thinking to facilitate transfer of knowledge.

“You have to think a little differently about the patient. You also need to be more focused on the summary being in-depth so it may be used at the conference. In that way, it can add a little extra bustle.” (R9)

Treatment changes could happen before the multidisciplinary conference as the information was passed on to the receiving outpatient clinics. Thereby, the information could affect patient care before meeting in person at the multidisciplinary conference, which could also lead to less discussion at the multidisciplinary conference.

“Sometimes, we come up with medical changes by meeting here. Not often, because often if I have written some thought about it might be a good idea to try with some sort of medicine, then it is effectuated at their next attendance.” (R9)

##### Theme 2) Knowledge sharing and collaboration

Interdisciplinary collaboration was already being practised. Respondents described existing easy access to knowledge sharing across disciplines e.g. reading the patient medical record or attending weekly medical conferences. However, benefits could be achieved from staging a formal collaboration. Initiatives to support decision-making were welcomed. All expressed unequivocal support to interdisciplinary collaboration, which they believed to make sense in many cases when promoting coordination of complex care. Moreover, the hospital was perceived as a specialised setting in terms of working across specialties. Several stated that the organisation should adjust to the patients, because the care services can benefit from collaboration across professions.

“Doctors do not always speak the same language. I often have questions about medicine (…) It is then clearly improved by talking together. Patient care can potentially be improved by this. I could easily get something out of it, but we are very used to sitting at conferences and talking interdisciplinary. This is a worship of the discipline; we are so good at.” (R4)

The respondents agreed that the healthcare professional who knows the patient should be the one to participate in a multidisciplinary conference. Therefore, the conference might include healthcare professionals outside the hospital. Some experienced that the discussions could depend on which professional groups participated. All stated that the conference did not take long. The overall impression was that attending the conference was prioritised. However, time and effort were valuable and should be used appropriately.

##### Theme 3) Selection of patients

Respondents experienced that some trajectories were parallel in their nature and that adjusting the inclusion criteria might target the intervention at the patients with most complexities. Several outpatient trajectories may not alone advocate for a multidisciplinary conference. Others expressed that the selection should consider indices, such as age, personal resources, and mental illness, i.e. taking a more holistic approach to selection. Consequently, many discussions during the conferences did not advance into long deliberations about the care, although, the conference was described as well led. The conference rarely contributed to new actions, as the care was often considered to be well-coordinated.

“Surprisingly, new actions are rarely taken. I would say that it often turns out that the treatment is fine and the treatments that run in parallel are actually quite well coordinated. I could imagine, some patient categories were better suited than others.” (R5)

#### Patients

##### Theme 1) Collective appointments

The patients appreciated that their appointments and tests had been aligned. This was stated by all respondents in different ways. However, three respondents also stated that things were also fine before the MOP, which made the MOP seem to matter less. They argued that being senior citizens, they had the spare time to attend multiple appointments. Some needed help from family members or friends to attend hospital appointments. Therefore, having multiple appointments scheduled on the same day was a good thing. One patient emphasised that coming to the hospital was mentally demanding and found it alleviating having appointments collected.

“Mentally, as soon as I must attend something like that, I hardly sleep a few days before and after (…). It was simply so great that I could do both on the same day. It requires a little more of me, mentally, because of a little waiting time between the appointment times, but it is definitely outweighed by the fact that it is one day.” (R8)

A feeling of security from being in outpatient trajectories, made patients want to continue their hospital attachment. One respondent realised that the MOP might led to reduced appointments or outpatient trajectories. Although this respondent thought many outpatient appointments were uncalled for, no attempts was made to communicate this as being in trajectories imbedded a feeling of security.

##### Theme 2) MOP was endurable

Respondents did not feel overwhelmed and reported no physical difficulties with spending half a day on outpatient appointments. This contrasted with their descriptions of own physical/mental capacity, e.g. having breathing problems and difficulties moving about. Longer time than usual was spent at the hospital, but only one patient expressed that the time felt too long. Nine respondents expressed that the waiting time between appointments was acceptable. While some considered it important to have limited waiting time, others did not emphasis this. Attending hospital outpatient appointments was described as a usual event. All responded that keeping track of information given during the day did not seem overwhelming, which was explained by familiarity with their diseases.

“I know my disease, so the talk is known. It’s nothing new they tell me. If it was a brand-new diagnosis, if they had told me, something completely new, then it might have been difficult to comprehend. But it is not so. I have had this for many years.” (R1)

##### Theme 3) Multidisciplinary decision-making

Patients were not invited to take part in the multidisciplinary conference, so their experiences were based on the notification they received from the care coordinators and on the response after the conference. Interdisciplinary collaboration on care services was valued, and the respondents appreciated that the MOP aimed to accommodate this.

“It would be a huge relief, when something new happened they would all know about it, instead of me having to inform them. I have many different symptoms and diseases and it could actually be nice to know if some of it was connected.” (R8)

A few of the patients did not recall being contacted after the conference. Therefore, these patients were aware that a multidisciplinary collaboration was taking place only because of the summon letter and the call from the care coordinators. Most patients felt no need to participate in the conference, as they feared feeling out of place in an academic medical discussion. From their experiences as patients, many technical terms were presented by the healthcare professionals.

“I see no reason to attend the conference because, even though I am fairly well-versed, I could imagine it taking place in technical terms, so you would sit and feel a little out-of-place. I know very well that the patient must be involved and have an influence on things. I feel that I have. (…) I am not afraid of the system.” (R6)

## Discussion

### Main findings

This study describes the formation and implementation of a tailored intervention to patients with multimorbidity with concurrent trajectories in outpatient clinics. The MOP includes patients based on their frequent attendance in outpatient clinics as identified by electronic records. Care coordination was challenged by context, although this hospital was focused on creation of integrated patient pathways. Navigating existing delivery systems, booking schemes, subspecialisation, and accommodating intentions to have patients followed by the same physician or nurse made care coordination challenging. During the first year, 102 patients underwent the MOP, corresponding to a reach of 20% of eligible patients. They were elderly patients, who at baseline assessed that follow-up/coordination could be improved, their physical well-being was low [56], and 43% reported medium/high treatment burden. Age and the combinations of chronic conditions resembled earlier intervention studies targeted patients with multimorbidity [33,61]. A total of 15% of all appointments were aligned and blood samples were reduced by 29%. In median, the patients used the intervention one time. The intervention was implemented as intended and conceptual elements were foremost followed accordingly. However, the handover of information in the transition between specialties lacked delivery. Although knowledge sharing already existed, healthcare professionals found a formal collaboration was welcomed but the patient selection required refinement. Patient respondents resembled the study population. They found the MOP endurable and appreciated having appointments and tests aligned. Furthermore, that multidisciplinary collaboration and regular visits to outpatient clinics provide a sense of security.

### Existing studies

Collaboration on care decisions is considered important for integration of patient care and includes sharing the responsibilities of problem solving and decision making [62]. Evidence on interventions aiming at ensuring multidisciplinary collaboration in a systematic manner, is lacking despite demands for integrated care solutions. Previous studies have no resemblance to the MOP [23,29,32,33,36,63,64,65,66,67], although programs have been identified in the ICARE4EU project [38]. An example is a holistic approach taken in Denmark. To support GPs, a multidisciplinary outpatient team offers a review of medications, care plan, and follow-up recommendations. Patients are selected on medical complexity, interacting diseases, and drug regimes [61], whereas the MOP includes patients based on their frequent attendance in outpatient clinics.

Concerns about care for patients with multimorbidity include disease-centred rather than patient-centred care, lack of attention to comorbidities, patient preferences and needs, and limited care coordination [23,35,68]. Reuben and Tinetti advocated for a wider span of conditions and more focused alignment of treatments toward common goals [69]. Likewise, Bayliss et al. have argued that an ideal process of care is also individualised to support the patient’s unique constellations of problems [28]. Moreover, Boye et al. have stated that healthcare systems need to create flexible systems for multimorbid patients [21]. The MOP accommodates many of these factors, although the approach to patient preferences and priorities are similar to the approach in usual care.

### Strengths and limitations

This is the first study to integrate outpatient appointments through intensified interdisciplinary collaboration on patients with multimorbidity. The study was performed in accordance with the well-established methods and prevailing guidelines, which ensured a systematic approach and transparency. Launching the intervention with five different outpatient clinics implied that different booking methods and work schedules could be equally managed, which gave a realistic idea of how a full-scale intervention will work. Data are presented on fidelity, reach, and acceptability and using mix-methods leads to a deeper understand of the intervention and is ideal to discover uncertainties in the intervention design and delivery [42,70]. The integration of methods was used when assessing fidelity.

Some limitations should be addressed. Missing data was a limitation. The time consumption among the care managers was difficult to document, we estimated it took one workday going through each monthly list of appointments. The same limitation applied to the treatment changes following MOP, which according to the interviews, seldom happened. Coordination was restricted to two weekdays. Additionally, CB being both interviewer and hospital employee may have affected responses and the analysis.

### Perspectives and implications

Despite similarities, patients with multimorbidity form a heterogeneous population with varying needs because conditions vary widely in their manifestations and treatment indications. Patient characteristics (treatment burden, physical well-being, and follow-up/coordination) underpin that these were patients with challenges. Despite this, both delivery system design/decision support and patient activation were assessed as good, which could point to that patient-centred care was already being delivered. Interviews revealed that the patient selection needs refinement because the conference discussions often did not have sufficient content or led to medical changes. This questions whether the intervention targets those most in need of multidisciplinary collaboration. However, alignment of hospital visits may seem relevant for some e.g., patients with mental disparities and patients who are reliant on help from caregivers. Complexity could be considered in the MOP, including condition severity, higher symptom burden, and polypharmacy to improve acceptability [71]. However, this might increase the work burden in the care model. Reach met our expectations, as appointments within the same speciality will have a time interval. Additionally, as intervals between appointments are not the same across specialties because they follow specific disease course programs, this limits how many appointments can be aligned and impact of the intervention.

The elements of the model are feasible and practicalities of organising the intervention are manageable and can be adapted, but this will require time, effort, and awareness of care coordination, patient information, and for healthcare professionals to engage. Self-booking and alignment of appointments by patients as well as adding voice-recognition for dictation summaries, might be considered, to ease hospital workload.

However, the organisational challenge of aligning appointments and setting up a multidisciplinary collaboration, as done in the MOP, must be addressed and efforts should be seen in relation to effectiveness. Our process-evaluation found system- and patient-level benefits gained by the MOP i.e., collaboration and knowledge-sharing across specialties, reduction of blood samples, acceptance of having appointments aligned, and a sense of collaboration among one’s healthcare professionals. Whether the MOP was effective in optimising treatment, led to reduced outpatient activity and fewer trajectories, or improved the patient-experience of care services and treatment burden, calls for investigation. Nonetheless, this study demonstrates the complexity of modifying patient trajectories to fit the existing organisational practices to integrate care for patients with multimorbidity.

## Conclusions

It was feasible to implement an intervention aligning outpatient appointments and integrating the care provided by different medical specialties. Yet, the intervention needs further development to better target patients with multimorbidity.

## Data Accessibility Statement

The datasets generated and/or analysed during the current study are not publicly available due to Danish law.

## Additional Files

The additional files for this article can be found as follows:

10.5334/ijic.6013.s1 Appendix 1. Modelling processes.

10.5334/ijic.6013.s2 Appendix 2. Patients in concomitant trajectories in outpatient clinics.

## Abbreviations

GP: General practitioner

IQR: Interquartile range

MOP: Multidisciplinary Outpatient Pathway

MRC: Medical Research Council

MTBQ: Multimorbidity Treatment Burden Questionnaire

PACIC: Patient Assessment of Chronic Illness Care questionnaire

PAS: Patient Administrative system

PROMS: Patient Reported Outcome Measures

SELFIE: Sustainable integrated chronic care models for multimorbidity – delivery, financing and performance

SF-12: Short-Form Health Survey (12 items)
