# Supplementary material for: Improving Health Care for Patients with Multimorbidity: A Mixed-Methods Study to Explore the Feasibility and Process of Aligning Scheduled Outpatient Appointments through Collaboration between Medical Specialties
Source: Int J Integr Care. 2022 Mar 1;22(1):17. doi: 10.5334/ijic.6013 (PMC8896239; doi:10.5334/ijic.6013)
Supplement: Appendix 1. — Modelling processes. [file ijic-22-1-6013-s1.pdf]

Appendix 1: Modelling processes

A wide span of hospital staff participated in designing, and included perspectives obtained from multimorbid patients, general practitioners (GPs), and municipality leaders. A steering group made the overall assessments, ensuring that all perspectives were carefully considered. Two working groups implemented the decisions made by the steering group, while ensuring that the solutions were feasible.

Involvement during the development of the Multidisciplinary Outpatient Pathway for multimorbid patients

Participants	Methods	Number*
Hospital task force team	Meetings – where the idea originated	Approx. 10 persons incl. Management, physicians, nurses, therapists, researchers, quality support officers.
Management for Regional Hospital Central Jutland	Meeting. Dissemination the protocol with acceptance to launch	2 managers
Centre management at Silkeborg Regional Hospital	Steering group:<br>Meetings, logbook with resumes, emails, informal discussions	1 chief nurse<br>1 chief physician<br>1 chief secretary<br>2 nurse ward managers
Patients	Focus group interviews:<br>Four groups with four patients, where incl. one caregiver, taking place at the hospital, audio-recorded, transcribed verbatim, systematic text condensation used in analysis.	16 patients<br>1 caregiver
General practitioners	Individual interviews:<br>1 individual face-to-face interview with a GP, who were also GPs' care coordinator: Audio recorded, transcribed verbatim, systematic text condensation.<br>3 individual telephone interviews: notes taken	4 GPs
Representatives from the municipality	Meeting with input to the intervention and discussion about the study population	2 primary care leaders representing the municipality
Nurse care coordinators	Steering group,<br>working groups,<br>meetings, logbooks with resumes,<br>emails, informal discussions	2 persons
Quality research officer		1 person
Researchers		3 persons
IT-supporter and data manager	Emails,<br>meetings,<br>informal discussions	2 persons
Secretaries/nurse administrators in charge of booking systems		Approx. 10 persons.
Work schedule coordinator for physicians	Emails, meetings,<br>informal discussions	1 person
Physicians	Steering group<br>Working groups<br>Meetings, logbooks with resumes, emails, informal discussions	9 physicians from different specialties
Nurses		3 nurses from different specialties
Laboratory manager	Emails, meetings, informal discussions	1 person, passing information to the rest of the laboratory

*The participants were involved at different times. The table sums the number of participants involved throughout the development.
