# Supplementary material for: Improving Health Care for Patients with Multimorbidity: A Mixed-Methods Study to Explore the Feasibility and Process of Aligning Scheduled Outpatient Appointments through Collaboration between Medical Specialties
Source: Int J Integr Care. 2022 Mar 1;22(1):17. doi: 10.5334/ijic.6013 (PMC8896239; doi:10.5334/ijic.6013)
Supplement: Appendix 2. — Patients in concomitant trajectories in outpatient clinics. [file ijic-22-1-6013-s2.pdf]

Appendix 2: Patients in concomitant trajectories in outpatient clinics

Register data showing outpatient activity was used to underpin decision making during development of the Multidisciplinary Outpatient Pathway.

Patients with two or more trajectories in outpatient clinics at Silkeborg Regional Hospital on 1 August 2018, including nine medical outpatient clinics*

	n	%
**Patients with trajectories in two or more outpatient clinics***	1,553	100
**Combination of specialties with highest number of outpatients***		
Cardiology – Pulmonology	311	20.0
Cardiology - Rheumatology	236	15.2
Pulmonology - Rheumatology	171	11.0
Cardiology – Gastroenterology	167	10.8
Endocrinology - Cardiology	137	8.8
**Number of trajectories in outpatient clinics per patient***		
2	1,376	88.6
3	154	9.9
>4	23	1.5

*Point prevalence on 1 August 2018 of patients with concomitant trajectories. All outpatient trajectories were assessed as to whether they were still active. The table includes all patients registered with two or more trajectories, also patients undergoing diagnostic testing.
